# Development and Evaluation of Rapid and Accurate CRISPR/Cas13-Based RNA Diagnostics for *Pneumocystis jirovecii* Pneumonia

**DOI:** 10.3389/fcimb.2022.904485

**Published:** 2022-06-15

**Authors:** Yangqing Zhan, Xiaoqing Gao, Shaoqiang Li, Yeqi Si, Yuanxiang Li, Xu Han, Wenjun Sun, Zhengtu Li, Feng Ye

**Affiliations:** ^1^ The First Affiliated Hospital of Guangzhou Medical University, Guangzhou Institute of Respiratory Health, State Key Laboratory of Respiratory Disease, National Clinical Research Center for Respiratory Disease, Guangzhou, China; ^2^ R&D Department, Hangzhou MatriDx Biotechnology Co., Ltd., Hanzhou, China

**Keywords:** transcription-mediated amplification, quantitative real-time PCR, infection, colonization, *Pneumocystis jirovecii* pneumonia

## Abstract

**Background:**

*Pneumocystis jirovecii* can result in a serious pulmonary infection, *Pneumocystis jirovecii* pneumonia, in immunocompetent hosts. The diagnosis of *Pneumocystis jirovecii* pneumonia has long been a major clinical concern, and there are limitations with the currently utilized immunostaining and polymerase chain reaction diagnosis/detection technologies (*e.g.*, insufficient sensitivity and accuracy). Hence, we sought to establish a rapid and RNA-specific transcription mediated amplification and CRISPR/Cas13a-based diagnostics targeted *P. jirovecii*-mitochondrial large subunit ribosomal RNA.

**Methods:**

The procedure of the diagnostics included amplification of the extracted RNA samples by transcription mediated amplification, followed by CRISPR/Cas13 detection, and ultimately, the judgment of the results after 30 minutes of fluorescence signal. Later, the diagnostic performance of the CRISPR/Cas13-based diagnostics were tested on the 62 surplus clinical samples.

**Results:**

This CRISPR/Cas13-based diagnostics achieved limits of detection of approximately 2 copies/µL transcribed RNA templates, with no cross reaction to other respiratory pathogens, including bacteria and fungi. Similar to in-house quantitative real-time polymerase chain reaction, CRISPR/Cas13-based diagnostics was still positive in 243-fold diluted bronchial alveolar lavage fluid. A preliminary evaluation of 62 surplus bronchial alveolar lavage fluid samples from patients suspected of *Pneumocystis jirovecii* pneumonia showed that CRISPR/Cas13-based diagnostics achieved a 78.9% sensitivity and a 97.7% specificity in the diagnosis of *Pneumocystis jirovecii* pneumonia.

**Conclusion:**

Our study demonstrates that the CRISPR/Cas13-based diagnostics technique has good performance for the accurate and specific diagnosis of *Pneumocystis jirovecii* pneumonia.

## Introduction

*Pneumocystis jirovecii* pneumonia (PJP) causes a serious disease burden on immunosuppressed hosts (Salzer et al., 2018). The mortality of PJP in immunosuppressed hosts, regardless of the status of HIV, was as high as 17-53% (Cordonnier et al., 2016). PJP was once the main complication and leading cause of death in AIDS patients (Djawe et al., 2015). It accounted for 36% of the mortality in HIV-infected patients (Gheibi et al., 2020). Since the application of antiretroviral therapy, the morbidity due to PJP has decreased significantly from 54.6% to 30.4%, with 5-year survival probabilities increasing from 1% to 69% and the mortality decreasing to 6.7% (Su et al., 2008; Djawe et al., 2015; Calvo-Lozano et al., 2020). However, PJP has been more prevalent in non-HIV immunocompromised patients than in HIV patients, with mortality up to 30%~50% (Su et al., 2008; Kanj et al., 2021).

Because *P. jirovecii* is extremely difficult to culture *in vitro*, visualization of characteristic cysts or trophozoites after the staining of respiratory specimens remains the gold standard to confirm PJP (Bateman et al., 2020). However, the positivity heavily relies on the quality of the specimen and the technician’s skill in microscopic examinations (Bateman et al., 2020; Chotiprasitsakul et al., 2020). At the same time, HIV-negative patients have a lower *P. jirovecii* load. The development of preventive medication has also led to a decrease in the *P. jirovecii* load. As a result, the sensitivity of *P. jirovecii* staining is as low as 31%. Therefore, the performance of *P. jirovecii* staining is unsatisfactory (Bateman et al., 2020).

Studies have shown that quantitative real-time PCR (qPCR) can quantitatively evaluate the load of *P. jirovecii* and improve the sensitivity when compared with *P. jirovecii* staining (Guegan and Robert-Gangneux, 2019; Alshahrani et al., 2020), and detection of *P. jirovecii* DNA by qPCR has been recommended for the mycological evidence of probable PJP (Donnelly et al., 2020). However, the cutoff to judge uncertain negative or positive readings associated with high Ct values varies among studies and populations (Cañas-Arboleda et al., 2019).

Another disadvantage of qPCR is that the target is DNA, which is not easily degraded due to its double-stranded structure. Evidence has shown that DNA amplification can remain positive for extensive periods of time (16 years for *Coxiella burnetiid* in Q fever endocarditis patients), despite effective bacterial killing (Keer and Birch, 2003; Edouard et al., 2013). It is not easy to distinguish a current infection from a previous infection *via* DNA by PCR (Edouard et al., 2013). The contamination of DNA in the laboratory can also contribute to false positive (Fang et al., 2018).

**Figure 1:**
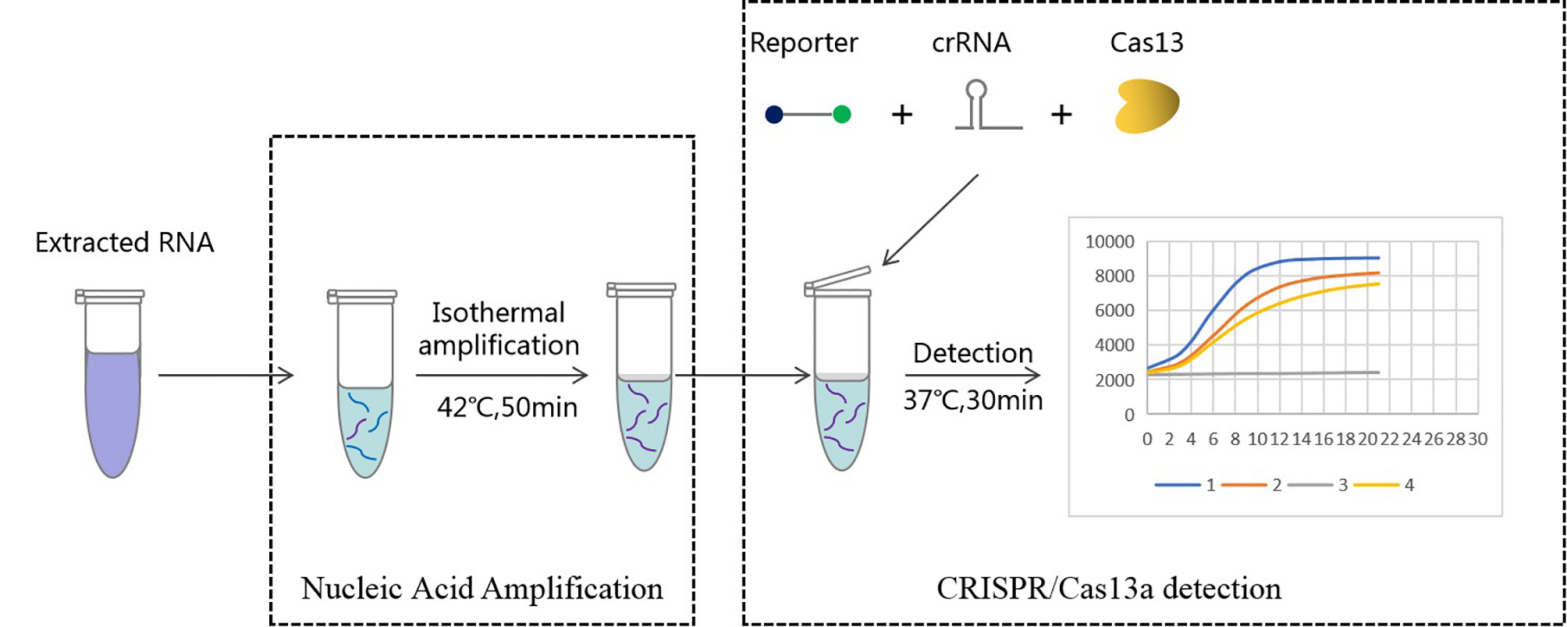
Workflow of Cas13a-based detection for *Pneumocystis jirovecii*. RNA extracted from bronchoalveolar lavage fluid was used as input for RapidCasD detection of *Pneumocystis jirovecii*. The results could be achieved within 60 minutes using a fluorescent reader detection method.

In this study, we established a rapid and RNA-specific transcription-mediated amplification (TMA) and CRISPR/Cas13a-based diagnostic (RapidCasD)-targeted *P. jirovecii*-mitochondrial large subunit ribosomal RNA (mtLSUrRNA). First, the extracted RNA samples were specifically amplified by TMA, and then the amplified products were detected by CRISPR/Cas13. Subsequently, fluorescence signals were used to analyze the sample test results on the fluorescent reader screen in less than 30 minutes (**Figure 1**). Ultimately, we evaluated the performance of RapidCasD and retrospectively applied this technique to test preserved bronchial alveolar lavage fluid (BALF) samples collected from patients with suspected PJP.

## Materials and Methods

### Preparation of the *P. jirovecii*-mtLSUrRNA RNA Template

Given that *P. jirovecii* is extremely difficult to culture, a *P. jirovecii* mtLSUrRNA positive control was generated. mtLSUrRNA of *P. jirovecii* is an attractive target for the molecular detection of PJP because of its highly repetitive sequence and has been widely used in the literature (Alanio et al., 2011; Tia et al., 2012). Hence, it was selected in our study.

The RNA template (SD-P.jir-mtLSUrRNA) of P. jirovecii-mtLSUrRNA that was used for TMA and the reverse RNA template (P.jir-mtLSUrRNA) of P. jirovecii-mtLSUrRNA that was used for the crRNA screening were synthesized by Nanjing Genscript Biotechnology Co., LTD. The copy number of RNA was calculated based on the quantitative concentration as determined by a micro spectrophotometer (Hangzhou ALLSHENG Instrument Co., Ltd., Nano300) using the following equation: RNA copy number = (M×6.02×1023)/(n×109×340), in which M represents the amount of RNA in nanograms, n is the length of the RNA in base pairs, and the average weight of a base is assumed to be 340 Daltons.

### Primer Design

**Figure 2:**
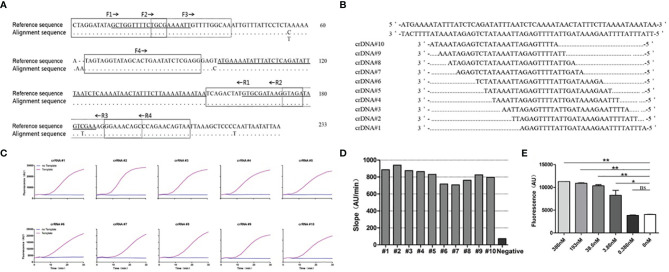
Design and screening of crRNAs for *P. jirovecii-*mtLSUrRNA. **(A)** Alignment of the mitochondrial large subunit ribosomal RNA locus (mtLSUrRNA) sequences of *P. jirovecii* (GenBank:MH010444.1) encompassing a 233-base-pair fragment. The reference sequence includes, but is not limited to, 8 sequences available in the GenBank database (accession numbers JF733749.1, KJ634837.1, KJ634823.1, KC470852.1, EU979566.1, KC470820.1, KC937059.1, KU693284.1). The locations of the primers for TMA are in broken lined or solid lined boxes and solid underscores, respectively. The locations of crRNAs for Cas13 are hyperbolically underlined. The dots represent identical nucleotides, and the nucleotides indicate occurrence in one or more sequences. **(B)** Ten different crRNAs were designed for a conserved region of the *mtLSUrRNA* gene. **(C)** crRNA screening. The reaction without template but with crRNA was used as a negative control for crRNA screening. **(D)** The results of the different crRNA screening reactions were normalized, and the ratio of the fluorescence value to time (Au/min) at 30 min was calculated. The crRNA #2 with the best performance, which has the highest slope (940) between 707 and 940, was used in subsequent experiments. **(E)** Bar graphs for the fluorescent signals for the Cas13a reaction obtained at 60 minutes. The data are presented as means ± SD (n = 3). Unpaired 2-tailed Student’s t tests were used to analyze the difference from 0 nM. The sensitivity of CRISPR/Cas13 was 3.86 nM, and the difference was significant (P =0.018). *P < 0.05, **P < 0.01, ns, non-significant.

TMA primer design: Aiming at the conserved region of the *P. jirovecii*-mtLSUrRNA gene, four forward primers and four reverse primers with the T7 promoter sequence were designed according to the justice chain sequence and the design principles of the TMA primers (**Figure 2**).

crRNA design: The crRNA sequence used in our study consists of two parts: one is the target region that specifically recognizes the RNA template, and the other region that binds the CRISPR/Cas13 protein is the sequence, gauuuagacuaccccaaaaacgaaggggacuaaaac (**Figure 2**). The target region of the crRNA sequences was designed from the antisense strand of the *P. jirovecii* conserved region on the CRISPR-RT website (http://bioinfolab.miamioh.edu/CRISPR-RT/). Then, we synthesized the crRNA sequences from Nanjing Genscript Biotechnology Co., Ltd.

### Nucleic Acid Extraction

According to the instructions of the Total Nucleic Acid Extraction Kit (Magnetic Bead Method) (Hangzhou Jieyi Biotechnology Co., Ltd., MD005), the reagents were added in turn to a deep-hole plate, and an automatic nucleic acid extraction instrument (Hangzhou Allsheng Instrument Co., Ltd, Autopure 20B) was used for nucleic acid extraction.

### Transcription Mediated Amplification Reactions

The TMA reactions were performed using a total volume of 5 µL, which was comprised of 0.5 µL amplified enzyme, 2.8 µL amplified buffer, 1 µM of each primer and 1.5 µL of RNA (predenaturated at 65°C for 5 min). Finally, 8 µL of mineral oil was added above the reaction surface. All reactions were incubated at 42°C for 50 minutes. In addition, the enzyme and buffer used in the reaction were all from Hangzhou Kasi Biotechnology Co., Ltd.

### Cas13 Detection Reaction

The CRISPR-Cas13a reaction included 2 µL of detection enzyme and 43 µL of detection buffer (Hangzhou Kasi Biotechnology Co., Ltd.), as well as 15 ng of crRNA probe and targeted RNA (*in vitro* transcribed RNA or TMA product). Then, the reaction was incubated in a fluorescence plate reader for 30 min at 37°C, and the fluorescent signals were collected every 30 seconds.

### qPCR

According to the instructions of the HiScript II U+ One Step qPCR Probe Kit (Nanjing Vazyme Biotechnology Co., Ltd., Q223-01) and the fluorescent quantitative PCR instrument (Xi’an TIANLONG Technology Co., Ltd., Gentier 96E), the procedure was as follows: predegradation at 95°C for 30 s, followed by 45 cycles at 95°C, 10 s, 60°C, and 30 s. The primers and probes used in this study were obtained from the literature and have obtained great significance in the clinical diagnosis of PJP (Mühlethaler et al., 2012).

### Droplet Digital PCR for the Quantification of the PJP Copy Numbers

In order to verify the relationship between the Ct value of qPCR and copies of *P. jirovecii*, a ddPCR assay was performed using the Sight DQ24 PCR System. The PCR mixture contained 0.4 μmol of each selected primer and 0.20 μM of each hybridization probe, 10 μL of Hieff Unicon^®^ Universal TaqMan multiplex qPCR master mix, 2 μL of purified DNA and approximately 10 μL of ddH_2_O to make a final reaction volume of 22 μL. The temperature profile for PCR was 60°C for 5 min and 95°C for 15 min, followed by 45 cycles of 95°C for 15 s, 60.0°C for 45 s and signal collection at 60°C for 1 min. The results were analyzed using SightPro software.

### Data Collection

Finally, in parallel with qPCR, we applied RapidCasD to test surplus BALF samples collected from HIV-negative patients with pneumonia. The surplus BALF samples that were associated with the respective patient’s clinical information were evaluated in the present study. If a patient had several samples at the same time, the earliest sample was selected for testing. The characteristics of the patients were retrospectively recorded by a medical chart review, and the collected data included the demographic data, clinical features and clinical diagnosis. The diagnosis of PJP was based on the definition from the European Organization for Research and Treatment of Cancer and the Mycoses Study Group Education and Research Consortium (EORTC/MSGERC) (Donnelly et al., 2020). Otherwise, it was considered colonization.

The diagnosis definition of PJP included patients with an underlying immunocompromised status, clinical signs of pneumonia, any consistent radiographic features and particularly ground glass opacities, consolidations, small nodules, infiltrate or cystic lesions in chest computed tomography, positive laboratory testing for *P. jirovecii* and the complete resolution of symptoms after a full course of anti-PJP treatment (trimethoprim-sulfamethoxazole). *P. jirovecii* colonization was defined as patients with a positive result in *P. jirovecii* nucleic acid tests but without the clinical features of PJP mentioned above or who recovered from pneumonia without anti-*P. jirovecii* treatment. An immunocompromised status was defined as the presence of one of the following diseases and/or treatment: solid organ or hematopoietic cell transplantation, neutropenia, solid or hematological malignancy under treatment, any types of known immunodeficiency disease, and other underlying disease in patients who were receiving more than 0.3 mg/kg.d systemic corticosteroids for more than 3 weeks within 60 days or any types of immunosuppressive drugs, such as methotrexate, azathioprine, cyclosporine, cyclophosphamide, etc.

The surplus BALF samples that were not associated with valid clinical information were excluded from the study.

### Statistical Analysis

In the repeated test results, the graphics were generated with GraphPad Prism 5.0 (GraphPad Software, Inc., San Diego, CA, USA). Unpaired 2-tailed Student’s t tests were used to analyze the differences in the quantitative data, and the chi-square test was used to compare the differences in the qualitative data.

The diagnostic performance of the RapidCasD and qPCR tests on the 62 surplus clinical samples were compared by the chi-square test. The sensitivity was calculated by the true positive/(true positive + false negative), the specificity was calculated by the true negative/(false negative + true negative), the positive predictive value was calculated by the true positive/(true positive + false positive), and the negative predictive value was calculated by the true negative/(true negative + false negative).

## Results

### The Design Region Selection and Screening for the crRNA Targeting of the mtLSUrRNA of *P. jirovecii*

We compared the mtLSUrRNA of 15 *P. jirovecii* strains and selected a conserved region to use as our detection target in the present study (**Figure 2A**). Ten crRNA sequences were designed for the crRNA screening (**Figures 2A, B**). The template-free reaction was used as the background control for the crRNA screening. All of the tested crRNAs showed specific activation upon mixing with the P.jir-mtLSUrRNA template, and the fluorescence signals increased with the detection time (**Figure 2C**). The slope of the ratio of the 30 min fluorescence value as compared to time was used as an index to analyze the performance of the crRNA. The crRNA #2, which had the best performance and had the highest slope (940) between 707 and 940, was used in subsequent experiments (**Figure 2D**).

Then, we tested the sensitivity of CRISPR/Cas13 with crRNA #2 and the RNA template (P.jir-mtLSUrRNA). Using unpaired 2-tailed Student’s t tests to analyze the fluorescence value difference at 30 min from the reaction of 0 nM showed that the sensitivity of CRISPR/Cas13 was 3.86 nM, and the difference was significant (P =0.018) (**Figure 2E**).

### Establishment of TMA for the mtLSUrRNA Amplification and Primer Screening

**Figure 3:**
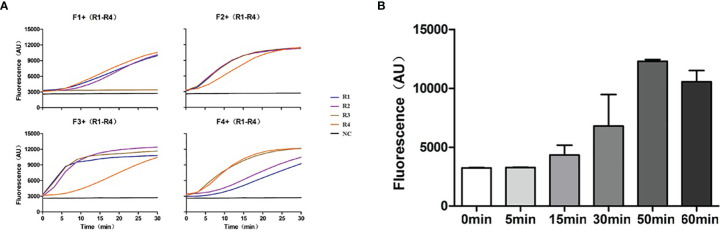
Primer screening and exploration of TMA amplification time. **(A)** The 16 paired primers were used for primer screening by TMA combined with the CRISPR/Cas13 detection method, and a primer-free reaction was used as the negative control. **(B)** The TMA products amplified at different times were detected by CRISPR/Cas13. TMA, Transcription Mediated Amplification.

As the detection sensitivity of CRISPR/Cas13 alone was not sufficient for PJP detection, we combined it with an isothermal amplification method to enhance the sensitivity of detection. At the same time, considering the specificity of RNA detection, TMA combined with the CRISPR/Cas13 detection method was selected to improve the detection sensitivity of PJP. According to the principle of primer design, four forward primers and four reverse primers were designed and paired to produce 16 pairs of primers for the primer screening. The amplified fragments included the specific recognition region of the crRNA. Among the 16 paired primers, except for F1+R3, the other primer pairs with 200 copies of RNA template (SD-P.jir-mtLSUrRNA) had obvious fluorescence detection signals compared with the negative control without template (NC) (**Figure 3A**). In addition, the paired primer F3+R1 with good performance was selected and was used in subsequent experiments for the detection of PJP.

To explore the optimal TMA amplification conditions, we compared the amplification effects at different time points with the primer pairs F3+R1 and crRNA #2. The results showed that the fluorescence value of CRISPR/Cas13 gradually increased with an increasing amplification time and reached the highest value when the amplification time was 50 min (**Figure 3B**). Therefore, an amplification time of 50 min was selected and was used for the subsequent experiments for the detection of PJP. At this time, a new detection assay system, named RapidCasD, which is a combination of TMA and CRISPR/Cas13, was successfully established.

### The Relationship Between the Ct Value of qPCR and Copy Number of ddPCR

The standard samples including 5 fg/uL, 1.67 fg/uL, 560ag/uL, 185ag/uL, 61.7ag/uL dilution were produced by using ddH2O for a 3-fold serial dilution of 15 fg/L linearized plasmid template. Both qPCR and ddPCR were used to calculate a standard curve and were analyzed by linear regression analysis. The Ct value by qPCR and copy number by ddPCR in five standard samples was plotted against the concentration (**Supplementary Figure S1**). Indeed, the R^2^ values of qPCR and ddPCR were 0.9998 and 0.9725, respectively, suggesting that the Ct value or copy number had a good linear correlation with the concentration of templates.

To show the relationship between the Ct value by qPCR and copy number by ddPCR, the data in **Supplementary Figure S1** were fitted and analyzed, the R^2^ value was 0.9762, and the linear regression curve was Y = -1.904*X + 38.35 (**Supplementary Figure S2**). Then, Ct value by qPCR for clinical performance in BLAF could be calculated.

### Performance Evaluation

**Figure 4:**
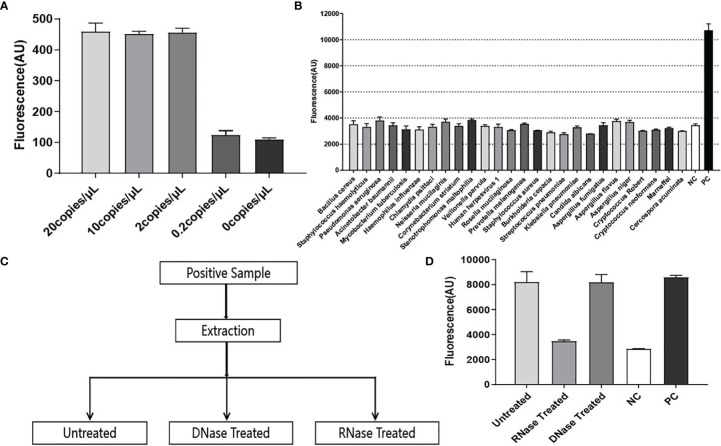
Performance evaluation of RapidCasD. **(A)** The lower limit of detection in the RNA template. Using the selected F3+R1 and crRNA #2, the ratio of the fluorescence value to time (Au/min) at 30 min (from 0–20 copies of SD-*P.jir*-mtLSUrRNA per µL) was measured, which was positive at 2 copies/µl; the mean ± SD (n = 2) of the slope value was used for the comparative analysis. **(B)** Pathogen specificity. The detection of a variety of bacteria, fungi and viruses showed good pathogen specificity. The data are presented as the means ± SD (n=2) of the fluorescence value at 30 min of detection and were used for the comparative analysis. **(C)** Sample processing flowchart during the RNA specificity verification. **(D)** RNA specificity. The reaction with 30 copies of SD-*P.jir*-mtLSUrRNA template was used as a positive control (PC), and the reaction without template was used as a negative control (NC). The data are presented as the means ± SD (n=3) of the fluorescence value detected for 30 min and were used for the comparative analysis.

To determine the analytical limit of RapidCasD, the transcribed RNA templates (SD-P.jir-mtLSUrRNA) were serially diluted at 20, 10, 2, and 0.2 copies/µL. Then, a 1.5 µL aliquot was extracted from each diluent sample as a template. The detection system for the target RNA template detected 2 copies/µL with a slope of 30 min of 457, which is much higher than the slopes of 0.2 copies/µL and 0 copies/µL at 30 minutes (**Figure 4A**). These results indicate that the RapidCasD assay we developed is capable of achieving a single-copy sensitivity.

To test the specificity of RapidCasD, samples from a clinical laboratory and from patients infected with common respiratory pathogens other than *P. jirovecii* were used for the specificity verification. The pathogens used for the specificity verification were as follows: *Bacillus cereus, Staphylococcus hemolyticus, Pseudomonas aeruginosa, Acinetobacter baumannii, Mycobacterium tuberculosis, Haemophilus influenzae, Chlamydia psittaci, Neisseria mucilaginis, Corynebacterium striatum, Stenotrophomonas maltophilia, Veillonella parvula, Rosella mucilaginosa, Prevotella melanogenes, Staphylococcus aureus, Burkholderia cepacia, Streptococcus pneumoniae, Kleebsiella pneumoniae, Candida albicans, Aspergillus fumigatus, Aspergillus flavus, Aspergillus niger, Cryptococcus Robert, Cryptococcus neoformans, Talaromyces marneffei, Cercospora acuminata and Human herpesvirus 1*. A reaction without the template was used as a negative control (NC), and a reaction with 30 copies of RNA template (SD-P.jir-mtLSUrRNA) was used as a positive control (PC). In this experiment, only the positive control was detected; the other common respiratory pathogen samples and negative control test results were negative (**Figure 4B**).

Due to the inevitable amplification of DNA nucleic acids, although the qPCR method can amplify RNA nucleic acid samples by reverse transcription, qPCR lacks RNA specificity. Hence, to prove that the RapidCasD assay in our study can only detect RNA but not DNA, the *P. jirovecii*-positive samples were divided into three equal parts. Then, two of them were treated with RNase or DNase, and one was untreated (**Figure 4C**). Nucleic acid was extracted from the processed samples and then was detected by the method we established. We found that fluorescence signals could be detected in both untreated and DNase-treated samples, which was higher than that of the negative control (NC) and was similar to that of the positive control (PC). However, there was no difference between the RNase treatment samples and the NC (**Figure 4D**). Therefore, the RapidCasD assay based on CRISPR/Cas13 that we established specifically targeted RNA but not DNA.

### Verification of RapidCasD in BALF

**Figure 5:**
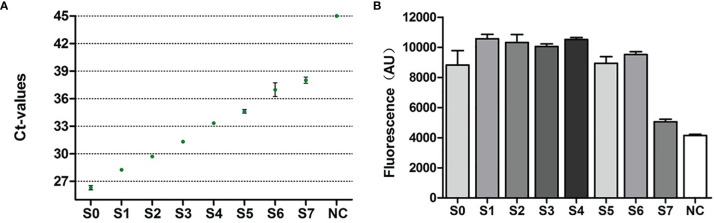
Performance characteristics of RapidCasD and qPCR in surplus BALF. **(A, B)** Limit of detection for qPCR and RapidCasD. The dots in the figure represent the Ct values of qPCR, and the column represents the fluorescence value of RapidCasD (30 min). A reaction without the template was used as the negative control.

Then, we compared the sensitivity of RapidCasD with the qPCR method in BALF samples. We serially diluted one PJP-positive BALF sample with a 3-fold gradient to obtain S1-S7 diluents. Then, the original solution (S0) and S1~S7 diluents were detected by qPCR and RapidCasD, respectively. The results showed that both the qPCR method and RapidCasD could stably detect S0 and its dilution S1-S7 (**Figures 5A, B**). Thus, the RapidCasD and qPCR methods have similar sensitivity in the testing of clinical samples.

**Figure 6:**
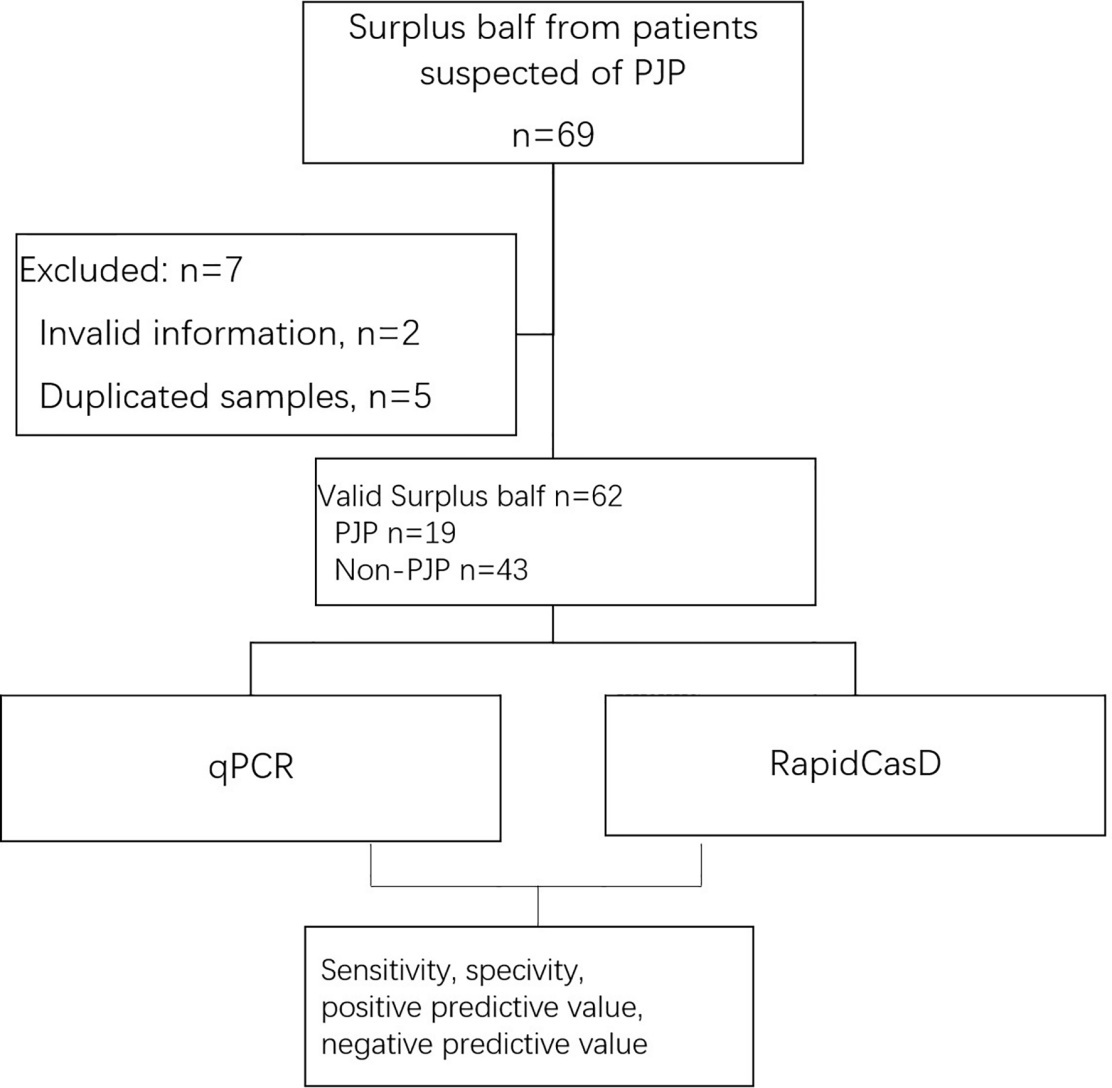
Workflow of the inclusion of the surplus BALF samples from patients suspected of having PJP.

Finally, in parallel with qPCR, we applied RapidCasD to 62 valid surplus BALF samples that were collected from 62 non-HIV patients at the First Affiliated Hospital of Guangzhou Medical University between April 2019 and April 2021 (**Figure 6**). Nineteen patients were diagnosed with PJP (**Table 1**), and they had a median age of 49.3 ± 15.7 years. Fifty-two patients (83.9%) were immunocompromised. The distribution of the immunocompromised patients included 10 patients who had solid organ or hematopoietic cell transplantations, 20 patients with solid or hematological malignancies under treatment, 21 patients who received immunosuppressive agents other than the above group and 1 patient with a known immunodeficiency disease. The underlying diseases were similar between the PJP and non-PJP patients, except that hematological malignancies were more common in the PJP patients (**Table 1**).

**Table 1:** Demographic data and comorbidities of the PJP and non-PJP patients.

Clinical Features	PJP	non-PJP	P value
Age (years)	49.3 ± 15.7	51.3 ± 15.5	0.641
Male/female	10/9	27/16	0.452
Smoker	1 (5.3%)	6 (14%)	0.319
Alcohol abuse	0 (0%)	2 (4.7%)	0.86
HSCT	0 (0%)	6 (11.6%)	0.212
Hematological malignancy	6 (31.6%)	3 (7.0%)	0.032
Solid organ transplantation	2 (10.5%)	2 (4.7%)	0.752
Solid malignancy	1 (5.3%)	10 (23.3%)	0.177
Anti-interferon-γ antibody deficiency syndrome	0 (0%)	1 (2.3%)	0.672
Interstitial lung disease	2 (10.5%)	6 (14%)	0.968
Other chronic lung disease	1 (5.3%)	5 (11.6%)	0.752
Diabetes mellitus	0 (0%)	5 (11.6%)	0.296
Coronary heart disease	0 (0%)	1 (2.3%)	0.672
Hypertension	4 (21.1%)	8 (18.6%)	0.901
Chronic renal disease	4 (21.1%)	5 (11.6%)	0.562
Chronic liver disease	0 (0%)	2 (4.7%)	0.86
Connective tissue diseases	3 (15.8%)	7 (16.3%)	0.744

The other chronic lung diseases include bronchiectasis, tracheal stenosis, and chronic obstructive pulmonary disease. HSCT, hematopoietic stem cell transplantation.

Compared with the non-PJP patients, expectoration was less common in the PJP patients, while the incidences of cough, dyspnea and fever were the same between the two groups. The PJP patients had a higher level of serum lactate dehydrogenase, higher positivity of 1-3-β-D-glucan, lower blood CD4 lymphocyte counts, and lower serum IgA values (P<0.05). The PJP patients were more prone to have lower levels of hemoglobulin and higher levels of blood urea nitrogen and creatinine; however, the differences were not significant. The most common findings in the images in the PJP patients were consolidation or patches, ground glass opacities, and cystic lesions, all of which are the same as those in the non-PJP patients (**Table 2**). Sixteen PJP patients received respiratory support, such as oxygen therapy and/or noninvasive ventilation, and this number was significantly higher than that in the non-PJP group. All of the PJP patients received trimethoprim-sulfamethoxazole treatment, and none of the non-PJP patients received anti-PJP treatment. Fortunately, neither the PJP nor non-PJP patients were admitted to the intensive care unit. All 62 patients recovered and were discharged from the hospital (**Table 2**).

**Table 2:** Comparison of the clinical features of the PJP and non-PJP patients.

Clinical features	PJP	Non-PJP	P value
Symptoms			
Fever	11 (57.9%)	17 (37.2%)	0.123
Cough	14 (73.7%)	38 (88.4%)	0.147
Expectoration	9 (47.4%)	35 (81.4%)	0.007
Hemoptysis	0 (0%)	3 (7.0%)	0.238
Dyspnea	12 (63.2%)	20 (46.5%)	0.227
Chest tightness/pain	4 (21.1%)	9 (20.9%)	0.991
Laboratory test			
Erythrocyte sedimentation rate (mm/1 h)	56.9 ± 28.5	64.0 ± 40.5	0.552
Procalcitonin (ng/ml)	0.23 ± 0.31	0.25 ± 0.29	0.826
White blood cell (^x^10^9/l)	8.23 ± 3.30	9.75 ± 5.09	0.25
Neutrophil (^x^10^9/l)	30.9 ± 35.2	35.9 ± 35.0	0.619
Lymphocyte (^x^10^9/l)	4.78 ± 6.57	8.01 ± 11.79	0.28
Hemoglobin (g/l)	98.7 ± 18.5	109.1 ± 23.1	0.097
Platelet (^x^10^9/l)	256.5 ± 114.3	282.4 ± 148.8	0.513
PaO2/FiO2	95.6 ± 27.4	110.4 ± 34.2	0.088
Alanine aminotransferase (U/l)	33.7 ± 62.3	24.8 ± 18.2	0.559
Blood albumin (g/l)	32.2 ± 3.96	32.8 ± 4.18	0.629
Aspartate aminotransferase (U/l)	26.2 ± 13.5	27.8 ± 27.1	0.822
Lactic dehydrogenase (U/l)	423.6 ± 183.2	263.2 ± 105.8	0.003
Blood urea nitrogen (mmol/l)	8.00 ± 5.53	5.15 ± 2.55	0.050
Blood creatinine (μmol/l)	104.8 ± 59.7	79.9 ± 27.1	0.105
D dimer (ng/ml)	1730.4 ± 1644.9	1411.0 ± 2089.4	0.584
Prothrombin time (s)	13.4 ± 0.86	13.9 ± 1.87	0.286
Fibrinogen (g/l)	3.41 ± 1.93	4.94 ± 2.15	0.013
Brain natriuretic peptide (pg/ml)	396.9 ± 598.4	379.4 ± 542.5	0.925
1,3-ß-D-glucan≥80 pg/mL	7 (36.8%)	5 (11.6%)	0.049
Positivity of *P. jirovecii* nucleic acid	19 (100%)	19 (44.2%)	<0.001
CD3+/CD45+ T lymphocyte (cells/μl)	595 ± 381.9	1104.3 ± 1010.2	0.074
CD3+/CD4+ T lymphocyte (cells/μl)	236.9 ± 123.0	480.5 ± 363.4	0.001
CD3+/CD8+ T lymphocyte (cells/μl)	282.1 ± 236.7	537.8 ± 794.1	0.262
T lymphocytes<50%	0 (0%)	3 (10%)	0.256
Th lymphocytes<29%	5 (38.5%)	12 (34.3%)	0.788
Ts lymphocytes<15%	1 (7.7%)	3 (8.6%)	0.922
Ratio of Th/Ts<1.4	10 (76.9%)	20 (57.1%)	0.208
B lymphocytes<5%	4 (33.3%)	13 (44.8%)	0.497
NK lymphocytes<2%	3 (25%)	9 (31.0%)	0.699
IL-2 (pg/ml)	1.28 ± 0.73	0.92 ± 0.74	0.101
IL-4 (pg/ml)	1.41 ± 0.90	1.22 ± 0.66	0.392
IL-6 (pg/ml)	16.0 ± 16.4	27.7 ± 52.9	0.392
IL-10 (pg/ml)	6.16 ± 4.37	4.03 ± 2.95	0.042
TNF-α (pg/ml)	1.69 ± 1.09	1.23 ± 0.98	0.138
TNF-γ (pg/ml)	4.79 ± 6.12	2.77 ± 7.85	0.363
IgG (g/l)	13.07 ± 8.60	12.13 ± 4.65	0.713
IgA (g/l)	1.33 ± 0.65	2.17 ± 1.27	0.004
IgM (g/l)	1.32 ± 1.51	0.99 ± 0.66	0.463
Radiological features			
Ground glass opacity	10 (58.8%)	14 (33.3%)	0.071
Interlobular thickening	3 (17.6%)	7 (16.7%)	0.828
Cystic lesions	5 (29.4%)	11 (26.2%)	0.801
Consolidation or patches	16 (94.1%)	39 (92.9%)	0.862
Plural effusion	1 (5.9%)	10 (23.8%)	0.109
Small nodules	2 (11.8%)	14 (33.3%)	0.091
CURB-65 score≥2	3 (16.7%)	4 (9.3%)	0.410
Respiratory support			0.002
No oxygen therapy	3 (15.8%)	25 (58.1%)	
Low flow oxygen therapy	10 (52.6%)	16 (37.2%)	
Median flow oxygen therapy	1 (5.3%)	2 (4.7%)	
High flow nasal oxygen	3 (15.8%)	0 (0%)	
Noninvasive ventilation	2 (10.5%)	0 (0%)	
Trimethoprim-sulfamethoxazole treatment	19 (100%)	0 (0%)	<0.001
Intensive care unit admission	1 (5.3%)	0 (0%)	0.672
Survival	19 (100%)	43 (100%)	1

**Figure 7:**
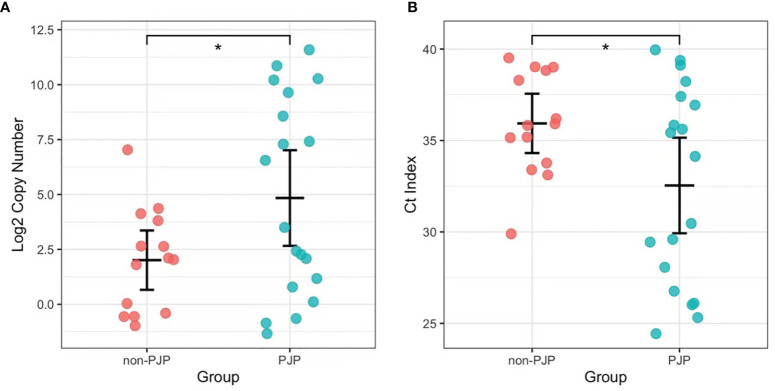
The load of *P. jirovecii* by qPCR in the PJP and non-PJP patients. Copy numbers (Log2, **A**) and Ct index **(B)** of *P. jirovecii* in the pjp AND non-PJP patients. *P < 0.05.

We found that 15 of the 19 BALF samples from the PJP patients were positive for *P. jirovecii* by RapidCasD, and all but 1 of the 43 BALF samples from the non-PJP patients were negative. All samples from the PJP patients were positive by qPCR, and 15 of 28 samples from the non-PJP patients were also positive. Thus, RapidCasD showed a perfect agreement with the clinical diagnosis of PJP compared to qPCR, with a sensitivity, specificity, positive predictive value and negative predictive value of 78.9%, 97.7%, 93.8% and 91.3% vs. 100%, 65.1%, 55.9% and 100% for qPCR, respectively, confirming the outstanding performance of RapidCasD to distinguish infection from colonization (**Table 3**). Because the non-PJP patients recovered without anti-*P. jirovecii* treatment, the positive *P. jirovecii* qPCR results in more than half of the patients in this group indicated colonization in the respiratory system. Although the bacterial load was higher in the PJP patients than in the *P. jirovecii*-colonized patients (4.72 ± 8.29*10^2 copies/µl vs. 0.15 ± 0.34*10^2 copies/µl, P=0.027), there was a great overlap between them (**Figures 7A, B**).

**Table 3:** Comparison of the performance of qPCR and RapidCasD using clinical samples.

	PJP	Non-PJP	Total
RapidCasD positive	15	1	16
RapidCasD negative	4	42	46
Total	19	43	62
	PPA:	NPA:	
	15 of 16 = 93.8%	42 of 46 = 91.3%	
qPCR positive	19	15	34
qPCR negative	0	28	28
Total	19	43	62
	PPA:	NPA:	
	19 of 34 = 55.9%	28 of 28 = 100%	

PPA, positive predictive agreement; NPA, negative predictive agreement.

**Figure 8:**
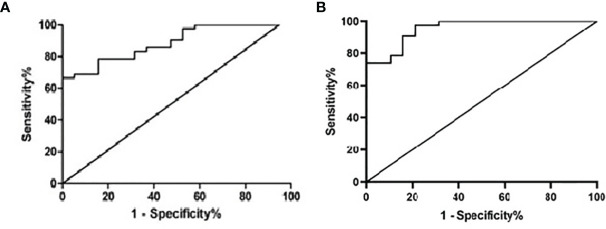
Receiver operator characteristic (ROC) curve of the qPCR and the RapidCasD for *P. jirovecii.* **(A)** Receiver operator characteristic (ROC) curve of the qPCR. **(B)** Receiver operator characteristic (ROC) curve of the RapidCasD.

According to the Youden Index (Specificity + Sensitivity-1), the sensitivity and specificity of the qPCR results of 62 clinical samples were analyzed, and the ROC curve analysis gave an area under the curve (AUC) of 0.886. When the Ct value for the qPCR was less than or equal to 40.05 (the corresponding template was approximately 4×10^2 copies/mL), the confidence interval was 0.8058~0.9661, the sensitivity was 100%, and the specificity was 66.67% (**Table 4**; **Figure 8A**). While, AUC of RapidCasD was 0.9536 (95%CI 0.9047~ 1.000, **Figure 8B**).

**Table 4:** Receiver operator characteristic (ROC) curve of the qPCR for *P. jirovecii* from 62 patients with *Pneumocystis jiroveci* pneumonia (PJP).

Gene	AUC	95%CI (%)	*P*	Sensitivity (%)	Specificity (%)
*MSG*	0.886	0.8058~0.9661	<0.0001	100	66.67

## Discussion

The basic principle of RapidCasD, a new RNA amplification detection method, is the combination of the RNA isothermal amplification technology with CRISPR/Cas13. RapidCasD has been proven to increase the sensitivity and to be comparable to the PCR method in our study. Additionally, RapidCasD achieved a higher specificity than PCR in discriminating between PJP and colonization. The excellent diagnostic performance of RapidCasD was considered to be based on the ability of CRISPR/Cas13 to recognize and cleave RNA, which improved upon the disadvantage of PCR, in which the target is DNA.

CRISPR technology was used for gene editing for the treatment of congenital disease in the early era. Gootenberg et al. first established a Cas13a-based molecular detection platform, termed Specific High-Sensitivity Enzymatic Reporter UnLOCKing, to detect specific strains of the Zika and Dengue viruses, to distinguish pathogenic bacteria, to genotype human DNA, and to identify mutations in cell-free tumor DNA (Srivastava et al., 2020). Later, CRISPR technology has been increasingly used in molecular diagnostics, including African swine fever virus (Wang et al., 2020) and tuberculosis (Lyu et al., 2020). However, it has not been used in the detection of *P. jirovecii*. Therefore, this is the first report in which CRISPR technology was successfully applied to the detection of *P. jirovecii*.

In contrast to DNA, RNA has a single-stranded structure and is easily degraded. It has good clinical relevance and is conducive to efficient evaluation and timely diagnosis. Different qPCR detection methods were used to quantitatively detect the copy number of different genes, including mtLSUrRNA, mtSSUrRNA, NAD1 and CYTB et al. And it was found that reverse transcriptase quantitative detection of RNA was more sensitive than other methods and targets (Valero et al., 2016; Dellière et al., 2021). When compared to RPA (recombinase polymerase amplification) and LAMP (loop-mediated isothermal amplification) which can only be used for DNA amplification, TMA technology is also reported to effectively amplify RNA (Ma et al., 2020). However, thi s RNA-specific amplification technique has not been previously reported to be applied in the detection of *P. jirovecii*. At the same time, the combination of the first introduction of CRISPR/Cas13a to detect the RNA of *P. jirovecii* further improved the specificity. Since there is no DNA product in the whole process, contamination and the false positive caused by DNA can be avoided. The sensitivity of RapidCasD to amplify RNA is comparable to that of PCR.

The load of *P. jirovecii* in the non-AIDS population with PJP is lower than that in AIDS patients, which results in a lower positivity of immunofluorescence staining and makes it difficult to distinguish infection versus colonization by qPCR (Jacobs et al., 2001; Piñana et al., 2020). In Piñana’s study, there was a large overlap in the threshold range of the Ct values between infection and colonization [29(26.4–34.7) vs. 34.6(30–41)] in hematological patients. It is extremely difficult to diagnose PJP in this group of patients solely according to the results of immunofluorescence staining or qPCR. The Ct value of *P. jirovecii* in the PJP patients in this study was even higher than that of the hematological patients in Piñana’s study, which indicated a lower load of *P. jirovecii*. Hence, in our study, PJP was diagnosed based on the comprehensive clinical features. Compared with the non-PJP patients, the indicators of *P. jirovecii* infection, such as elevated 1,3-ß-D-glucan, LDH and CD4+ T lymphopenia, were more common in the PJP group. Ground glass opacities, interlobular thickening and small nodules on chest CT scan were more common in the PJP group, although there was no statistical significance between the two groups. All of the PJP patients recovered after anti-pneumocystis treatment, while the non-PJP patients recovered without anti-pneumocystis treatment. Hence, to a certain extent, the grouping of cases in this study reflects the real status of *P. jirovecii* infections in HIV-negative patients who were evaluated in the present study.

Colonization has always been a difficult problem in PJP diagnosis. There is currently a lack of effective and sensitive methods to distinguish infection from colonization. The disadvantage of the staining method as the diagnostic gold standard is its insufficient sensitivity and cumbersome operation (Alshahrani et al., 2020). While a positive PCR result could be difficult to interpret, as the thresholds for distinguishing between colonization and infection has varied among methods, studies and populations (Azoulay et al., 2009; Mühlethaler et al., 2012; Hoarau et al., 2017; Perret et al., 2020; Piñana et al., 2020). The upper cutoff value of RT–PCR for infection in some studies was set at 1*10^3 copies/ml for HIV-negative patients, while it was 3.2*10^6 copies/ml for FTD (Hoarau et al., 2017). In Mühlethaler’s study, a lower limit of 1,450–1,900 pathogens/mL-1, as detected by quantitative PCR, showed good performance in diagnosing definitive PJP when compared to the immunofluorescence methods. However, the similarity between PCR and FTD is that there is a large gray zone in the results, and sometimes the results cannot distinguish between colonization and infection. In the present study, RapidCasD diagnosed *P. jirovecii* infection with a specificity of 97.7%, which was superior to qPCR, showing the potential of this method to distinguish infection from colonization. However, further verification in prospective studies is needed.

This study shows the perfect diagnostic performance of RapidCasD in BALF. However, this technique requires an invasive procedure. As a noninvasive specimen, sputum had been shown to be an alternative specimen type for patients in whom BALF cannot be obtained (Zhan et al., 2021). Additionally, studies have shown that other types of specimens, such as oral washes, throat swabs, and blood samples, can be used to diagnose PJP (Goterris et al., 2019). Hence, the application of RapidCasD needs to be explored in these noninvasive sample types to improve the diagnosis of PJP in seriously ill patients.

Another limitation of our study is that only preserved surplus samples were tested. It is possible that RNA may degrade with the extension of the storage time or with nonproper preservation, leading to a reduced sensitivity. Additionally, only part of the patients enrolled in this study received non-invasive ventilation, and one patient was admitted to the ICU, which led to the fact that the patients in this study were mainly mild. This is indeed a limitation. A possible factor to consider is that we have increased our vigilance against the occurrence of PJP in immunosuppressed hosts, enabling early diagnosis and treatment, and improving prognosis. Moreover, the number of specimens in this study is small. Our new CRISPR/Cas13a technology needs to be verified in a larger sample size later. Ultimately, the samples in this study were taken from non-HIV patients, so the result is only applicable to this group of patients.

## Conclusion

In the present study, we established a TMA and CRISPR/Cas13a-based Diagnostics (RapidCasD) technique that targets RNA and shows good performance for an accurate and specific diagnosis of PJP using a short turnout time.

## Data Availability Statement

The raw data supporting the conclusions of this article will be made available by the authors, without undue reservation.

## Ethics Statement

The studies involving human participants were reviewed and approved by Ethics committee of the First Affiliated Hospital of Guangzhou Medical University. The patients/participants provided their written informed consent to participate in this study.

## Author Contributions

Conceptualization, YZ and XG; methodology, XG and YS; software, XG; validation, YS; formal analysis, SL and YL; investigation, YL and WS; resources, YZ; data curation, XG, YL, WS and ZL; writing—original draft preparation, YZ and XG; writing—review and editing, YZ and XH, FY; supervision, FY; project administration, YZ; funding acquisition, YZ. All authors contributed to the article and approved the submitted version.

## Funding

This work was sponsored by the grant of State Key Laboratory of Respiratory Disease (SKLRD-Z-202224).

## Conflict of Interest

Authors XG, YS, and XH are employed by Hangzhou MatriDx Biotechnology Co., Ltd. China.

The remaining authors declare that the research was conducted in the absence of any commercial or financial relationships that could be construed as a potential conflict of interest.

## Publisher’s Note

All claims expressed in this article are solely those of the authors and do not necessarily represent those of their affiliated organizations, or those of the publisher, the editors and the reviewers. Any product that may be evaluated in this article, or claim that may be made by its manufacturer, is not guaranteed or endorsed by the publisher.

## Supplementary Material

Supplementary Figure S1 Performance of qPCR and ddPCR techniques on standard samples produced from a linearized plasmid template. The qPCR and ddPCR techniques were performed on 5 standard samples produced from a 15 fg/L linearized plasmid template. The Ct value by qPCR **(A)** and copy number by ddPCR **(B)** of the five standard samples (5 fg/L, 1.67 fg/L, 560ag/L, 185ag/L, 61.7ag/L dilution) were analyzed against the concentration

Supplementary Figure S2 The linear relationship between the Ct value by qPCR and copy number by ddPCR.
