# Supplementary figures and images for: Development and Evaluation of Rapid and Accurate CRISPR/Cas13-Based RNA Diagnostics for Pneumocystis jirovecii Pneumonia
Source: Front Cell Infect Microbiol. 2022 Jun 15;12:904485. doi: 10.3389/fcimb.2022.904485 (PMC9240425; doi:10.3389/fcimb.2022.904485)

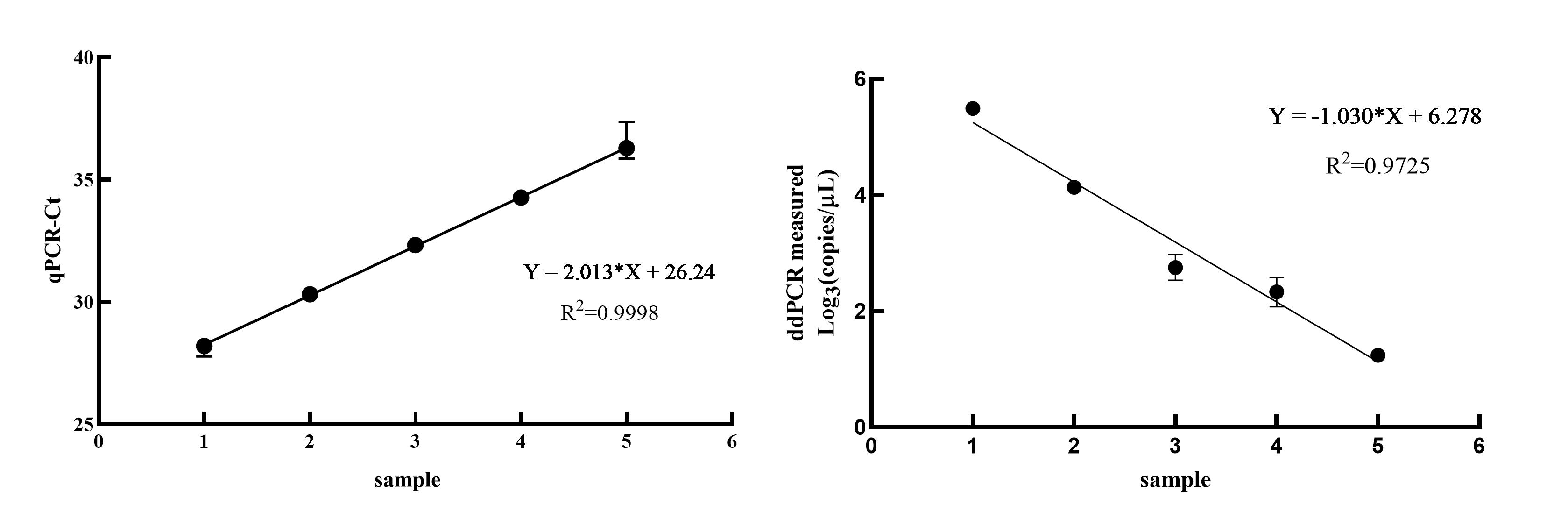

Supplement: Supplementary Figure S1 — Performance of qPCR and ddPCR techniques on standard samples produced from a linearized plasmid template. The qPCR and ddPCR techniques were performed on 5 standard samples produced from a 15 fg/L linearized plasmid template. The Ct value by qPCR (A) and copy number by ddPCR (B) of the five standard samples (5 fg/L, 1.67 fg/L, 560ag/L, 185ag/L, 61.7ag/L dilution) were analyzed against the concentration [file Image_1.jpg]

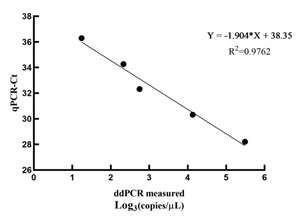

Supplement: Supplementary Figure S2 — The linear relationship between the Ct value by qPCR and copy number by ddPCR. [file Image_2.jpeg]
